# Intelligent UHF Sensor-Based Partial Discharge Fault Diagnosis in GIS Using a Temporal-Frequency Dual-Branch Stochastic Configuration Network

**DOI:** 10.3390/s26185739

**Published:** 2026-09-09

**Authors:** Mingyuan Hu, Jingwen Liu, Baolong Yu, Ying-Ren Chien, Lei Zhang

**Affiliations:** 1 School of Electrical Engineering, Shandong Huayu University of Technology, Dezhou 253000, China; hmy@huayu.edu.cn (M.H.); ljw@huayu.edu.cn (J.L.); 2 School of Mechanical and Electrical Engineering, Jiujiang Polytechnic University of Science and Technology, Jiujiang 332007, China; hrbybl@163.com; 3 Department of Electronic Engineering, National Taipei University of Technology, Taipei City 10608, Taiwan; 4 School of Electrical Engineering, Beijing Jiaotong University, Beijing 100091, China; 22110464@bjtu.edu.cn

**Keywords:** GIS, partial discharge pattern recognition, stochastic configuration network, temporal-frequency features, intelligent sensors, power equipment fault diagnosis, process innovation

## Abstract

Gas-insulated switchgear (GIS) is an important component of power transmission systems. Accurate partial discharge (PD) pattern recognition is a key requirement for identifying internal insulation defects within the equipment. However, ultra-high-frequency (UHF) PD pulse sequences produced by different insulation defects usually contain complex nonlinear temporal structures and multi-scale periodic variations. These coupled characteristics are difficult to describe adequately via a single feature-mapping strategy. Thus, this paper proposes a temporal-frequency dual-branch stochastic configuration network (TF-SCN), which consists of two heterogeneous hidden-layer branches, for GIS PD pattern recognition. Specifically, in the temporal branch, the model uses a non-periodic, nonlinear activation function similar to that used in a conventional SCN to capture the nonlinear temporal characteristics. The frequency-sensitive branch introduces paired sine–cosine harmonic nodes with shared random projection parameters to capture frequency-sensitive features. The hidden outputs of the two branches are concatenated into a joint temporal-harmonic feature space, and the output weights are solved under the residual inequality constraints for GIS PD classification. To verify the superiority of the proposed model, comparative experiments are conducted on a dataset containing four PD patterns collected from the GIS PD experimental platform. Several baseline models, including 1DCNN, BPNN, SVM, KELM, RVFL, and SCN, are selected for performance comparison. The results show that, compared to 1DCNN, BPNN, SVM, KELM, RVFL, and SCN, TF-SCN effectively extracts distinguishable features in both the time and frequency domains, thereby achieving the best overall performance. Furthermore, its recognition performance remains consistently superior even on noisy data with signal-to-noise ratios ranging from 50 dB to 20 dB. By integrating highly sensitive UHF sensors with the proposed TF-SCN, this study presents a robust, AI-enhanced intelligent sensing and fault diagnosis system for continuous condition monitoring of power equipment.

## 1. Introduction

Gas-insulated switchgear (GIS) has been widely used in power transmission systems because of its compact structure and reliable operation. Nevertheless, internal insulation defects may arise during manufacturing, installation, or long-term operation. Typical defects include metal tips, voids inside epoxy insulation, floating potentials, and free metal particles. These defects tend to trigger partial discharge (PD) [1,2,3,4] under a strong electric field. PD activity further accelerates the degradation of insulating materials, ultimately leading to insulation breakdown [5,6]. Therefore, identifying PD defect patterns accurately is important for assessing the insulation condition of GIS and supporting maintenance decisions.

Ultra-high-frequency (UHF) detection serves as an essential method for GIS PD monitoring because of its high sensitivity and strong resistance to electromagnetic interference [7,8]. PD pulses generated by different insulation defects vary in occurrence time, event density, and amplitude distribution, since the discharge process depends on the local electric field, carrier motion, and defect geometry. Traditional PD pattern recognition usually relies on phase-resolved partial discharge (PRPD) maps [9] or manually designed statistical features [10], followed by classifiers, such as support vector machines (SVM) [11,12] or back-propagation neural networks (BPNN) [9]. These models achieve favorable results under ideal conditions, but their effectiveness depends largely on the predefined input features. Such handcrafted features are generally insufficiently detailed to accurately reflect the nonlinear pulse sequence behavior of PD events, particularly under conditions of high noise and variable operating conditions. To elevate conventional UHF detection into an intelligent sensing system, it is crucial to equip the sensors with advanced AI-enhanced signal processing algorithms capable of robust fault diagnosis.

Deep learning methods, especially convolutional neural networks, have attracted considerable attention in PD pattern recognition because of their strong nonlinear feature extraction ability [13,14]. They are able to learn high-dimensional temporal features directly from the raw signals, thereby reducing the reliance on handcrafted features [15,16,17,18]. However, this advantage is accompanied by increased model complexity. Deep learning models usually contain a large number of trainable parameters and require large-scale training data as well as iterative optimization [19,20]. As a result, their training cost is relatively high, and their deployment demands substantial computational resources in field monitoring devices. Recently, deep learning-based intelligent fault diagnosis technologies have been widely applied in research on various industrial systems. By integrating heterogeneous information with advanced feature learning strategies, these methods demonstrate significant potential for improving diagnostic accuracy and interpretability under complex operating conditions [21,22,23]. Moreover, strategies for integrating physical constraints have become an effective technical approach for improving fault diagnosis performance under complex field conditions [24,25,26,27]. In practical conditions, available defect samples are usually limited, and the measured signals are easily affected by environmental noise and variations in measurement conditions. Therefore, it is necessary to develop a PD recognition model with a small parameter count and strong feature-representation capability.

Random neural networks provide an efficient alternative for fast PD pattern recognition under limited-sample conditions. Random learning models, such as extreme learning machine (ELM) and random vector functional-link network (RVFL), randomly assign the input weights and biases of hidden nodes, and then solve the output weights by least squares. This learning scheme is simple to implement, computationally efficient, and fast to train. However, its drawbacks are also evident: its performance and stability still require further improvement, as the hidden parameters are generated without residual-guided supervision. Thus, Wang et al. [28] proposed the stochastic configuration network (SCN) based on random basis function approximation and universal approximation theory. SCN introduces residual inequality constraints during the incremental construction of hidden nodes. The current training residual acts as supervisory information, and only candidate nodes that satisfy the residual convergence requirement are accepted. This mechanism improves the effectiveness of random node configuration while preserving the analytical solution of the output layer [29]. SCN therefore provides a promising framework for efficient GIS insulation defect diagnosis.

However, the feature representation capability of conventional SCN remains constrained by its simple network structure and the form of random basis functions. Most SCN models construct the hidden feature space using a single nonlinear activation function, such as sigmoid or tanh. For UHF PD pulse sequences with repetitive characteristics, this is insufficient. Different PD patterns not only differ in terms of pulse time distribution and amplitude variations, but also exhibit distinct multiscale frequency responses. A single nonlinear mapping struggles to capture both temporal structure and frequency variations simultaneously. This limits the ability of SCN to extract discriminative information from different pulse sequences.

Hence, this paper proposes a temporal-frequency dual-branch stochastic configuration network (TF-SCN) for PD pattern recognition. The model receives the PD UHF pulse-event sequence as the network input and constructs two branches in the hidden layer: a temporal feature branch and a frequency-sensitive feature branch. The temporal branch applies conventional nonlinear stochastic configuration nodes to map pulse sequences and describe differences in pulse timing, amplitude variation, and local event distribution. The frequency-sensitive branch introduces paired sine–cosine harmonic nodes with shared random projection parameters. These periodic basis functions provide a frequency-sensitive response in the random feature space. Subsequently, the heterogeneous hidden features from these two branches are concatenated, enabling the model to jointly represent both the temporal structure and frequency-sensitive information in the PD event sequence. The main contributions of this paper are summarized as follows:

(1) A temporal–frequency dual-branch stochastic configuration network (TF-SCN) is proposed for GIS PD pattern recognition, where temporal nonlinear mapping and harmonic frequency-sensitive mapping are integrated to enhance the feature representation capability of SCN.
(2) A heterogeneous feature-space construction strategy is introduced into SCN through basis-function modifications, combining temporal nonlinear nodes and shared sine–cosine harmonic nodes to provide complementary temporal and frequency-sensitive representations.
(3) Comprehensive experiments, including comparative analysis, ablation studies, and noise robustness tests, are conducted to verify the effectiveness and stability of the proposed TF-SCN for GIS PD pattern recognition.

The remainder of this paper is organized as follows. Section 2 introduces the proposed TF-SCN framework. Section 3 describes the experimental design, including data preparation, evaluation metrics, and comparative experiments. Section 4 presents the experimental results and discussion. Finally, Section 5 summarizes the main conclusions and discusses future research directions.

## 2. PD Pattern Recognition Model Based on TF-SCN

### 2.1. Overall Framework of the Proposed Method

**Figure 1:**
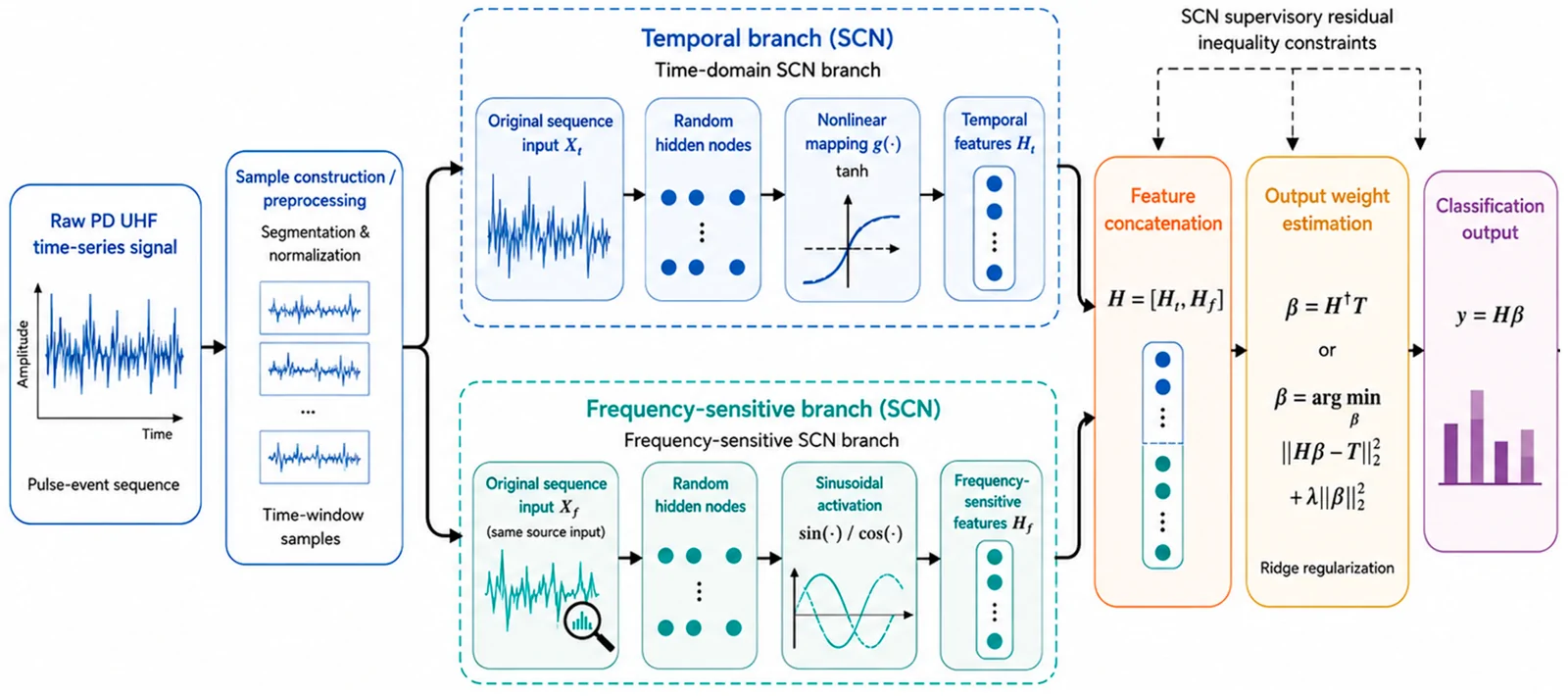
Architecture of the proposed TF-SCN.

Conventional methods, such as SCN, generally construct the hidden feature space with a single type of random basis function. This poses challenges in simultaneously representing the nonlinear temporal and frequency variations in PD impulse sequences. Therefore, this paper proposes TF-SCN for GIS PD pattern recognition. As shown in Figure 1, the model mainly consists of sample construction and normalization, temporal-frequency feature mapping, feature-level concatenation, and analytical output-layer solution.

First, the *i*-th PD UHF pulse-event sequence, after time-window segmentation and normalization, is denoted as:

(1) $$x_{i}=\left[x_{i,1},x_{i,2},\ldots ,x_{i,d}\right]\in R^{d}$$

where *d* is the input dimension; pulse event $x_{i,k}=[t_{i,k},s_{i,k}]\;\in R^{2}$ c consists of pulse time $t_{i,k}$ and amplitude $s_{i,k}$. Thus, a dataset containing *N* samples is denoted as $X=[x_{1},x_{2},\ldots ,x_{N}]\;\in \;R^{N\times d}$, and the corresponding one-hot label matrix is $Y=[y_{1},y_{2},\ldots ,y_{N}]\in R^{N\times C}$, where C represents the number of PD patterns.

Then, both the temporal SCN branch and the frequency-sensitive SCN branch receive the same PD UHF pulse sequence X as input. The temporal branch utilizes traditional nonlinear random configuration nodes to represent temporal variations in pulse distribution and amplitude variations. On the other hand, the frequency-sensitive branch employs pairs of sine–cosine harmonic nodes with shared random projection parameters. These periodic basis functions enhance the ability of the model to respond to oscillations and frequency-dependent variations.

Finally, when both branches have performed feature mapping, the time-domain features and frequency-sensitive features are concatenated to form a heterogeneous joint representation. Subsequently, the output weights are obtained via a least-squares solution, and the PD defect category is determined based on the maximum output response. Therefore, TF-SCN retains the lightweight training advantages of SCN while further enhancing the model’s ability to jointly represent time-domain and frequency-sensitive information in PD pulse sequences.

### 2.2. Temporal Branch Nodes

The temporal branch is designed to capture nonlinear temporal features within pulse sequences. PD events caused by different GIS insulation defects exhibit distinct patterns in terms of their temporal distribution and amplitude variations. These differences provide important information for defect identification. For the *j*-th temporal stochastic configuration node, the input weight and bias are randomly generated. The feature representation of this node for the training samples is written as:

(2) $$g_{j}^{T}=tanh(X\cdot w_{j}^{T}+b_{j}^{T})$$

where tanh(·) is the hyperbolic tangent activation function, $w_{j}^{T}$ and $b_{j}^{T}$ are the input weight and bias of the temporal node.

### 2.3. Frequency-Sensitive Branch Nodes

Besides temporal differences, the impulse sequences of different PD events also exhibit variations in oscillatory behavior and multiscale frequency responses. Conventional SCNs commonly adopt non-periodic activation functions, such as the S-function or the tanh function. Although these functions possess nonlinear approximation capabilities, they lack the ability to explicitly enhance the response to periodic and frequency-dependent patterns. Therefore, the proposed model introduces pairs of sine–cosine harmonic nodes in the frequency-sensitive branches. This enables the random feature space to exhibit periodic responses that differ from the traditional nonlinear mappings. Notably, this branch avoids explicitly using spectral features as input. It continues to utilize the original UHF pulse sequence, deriving its frequency sensitivity from the random mapping of periodic basis functions in the hidden layers.

In this branch, the shared input weights $w_{j}^{F}$ and biases $b_{j}^{F}$ are the configuration parameters of the *j*-th harmonic node. This results in a stochastic projection $z_{j}^{F}=X\cdot w_{j}^{F}+b_{j}^{F}$. On this basis, a pair of harmonic nodes is constructed as:

(3) $$g_{j}^{F}=sin(z_{j}^{F})+cos(z_{j}^{F})$$

where $g_{j}^{F}$ denotes the latent feature representation produced by the j-th harmonic node pair. Since the sine and cosine components share the same random projection parameters, they describe complementary phase information for the same implicit periodic response. When a phase disturbance occurs in the implicit periodic response, i.e., $z_{j}^{F}\to z_{j}^{F}+∆$, the paired harmonic response becomes:

(4) $$g_{j}^{F}\left(z_{j}^{F}+∆\right)=\left[\begin{array}{cc} \sin\left(z_{j}^{F}\right) & \cos\left(z_{j}^{F}\right) \end{array}\right]\left[\begin{array}{cc} \cos\left(∆\right) & -\sin\left(∆\right)\\ \sin\left(∆\right) & \cos\left(∆\right) \end{array}\right]\left[\begin{array}{c} 1\\ 1 \end{array}\right]$$

This relation shows that the paired sine–cosine nodes form a phase-complete two-dimensional harmonic representation space. When a phase shift occurs, the paired output mainly appears as a rotation in this harmonic subspace. This reduces the sensitivity of a single periodic basis function to phase disturbance. Moreover, in the frequency-sensitive branch, benefiting from shared projection parameters, the model captures both frequency and phase information through sine and cosine functions. This paired structure enables the hidden layer to characterize periodic oscillations and frequency-dependent variations from different PD pulse sequences without the manual selection of frequency components. Compared to independent random parameters, the shared projection strategy both maintains consistency in harmonic responses and enhances the ability of the network to capture intrinsic frequency-sensitive patterns.

### 2.4. Feature Concatenation and Output-Layer Solution

Two latent feature matrices are generated by the temporal branch and the frequency-sensitive branch: the temporal feature matrix $H_{T}=[g_{1}^{T},g_{2}^{T},\ldots ,g_{L_{T}}^{T}]\in R^{N\times L_{T}}$ and the frequency-sensitive feature matrices $H_{F}=[g_{1}^{F},g_{2}^{F},\ldots ,g_{L_{F}}^{F}]\in R^{N\times L_{F}}$. The former describes temporal distribution, amplitude variations, and local temporal information, while the latter enhances the perception of periodic oscillatory variations. To enhance the feature perception capabilities of the model, feature-level concatenation is applied to construct a joint latent representation:

(5) $$H=[H_{T},H_{F}]\in R^{N\times (L_{T}{+L}_{F})}$$

In the current hidden layer structure, the residual $E=[e_{1},e_{2},\ldots ,e_{C}]$ between the model prediction and the actual label is:

(6) E=Y−Hβ

where *β* is the output weight matrix. To reduce the risk of overfitting, the output weights are obtained by minimizing an L2-regularized residual:

(7) $$\beta ={\left(H^{T}H+\lambda I\right)}^{-1}H^{T}Y$$

where *λ* is the regularization coefficient and *I* is the identity matrix.

Similar to the original SCN, the residual produced by the current network structure informs the generation of the hidden-node weights and biases at the next hidden node. The supervisory residual constraint is expressed as:

(8) $$\{\begin{array}{c} \varepsilon_{q}^{T}=\frac{{<e_{q},H_{T}>}^{2}}{<H_{T},H_{T}>}-\left(1-r-\mu_{L}\right){\left\|e_{q}\right\|}_{2}^{2}\geq 0,q=1,2,\ldots ,C\\ \varepsilon_{q}^{F}=\frac{{<e_{q},H_{F}>}^{2}}{<H_{F},H_{F}>}-(1-r-\mu_{L}){\left\|e_{q}\right\|}_{2}^{2}\geq 0,q=1,2,\ldots ,C \end{array}$$

where $e_{q}$ is the residual of the q-th class under the current hidden-layer structure, r∈[0,1] is the contraction parameter, and $\mu_{L}$ is a non-negative decreasing sequence. A temporal node or a frequency-sensitive node is added to the corresponding branch only when it satisfies the above inequality for all output dimensions. Notably, the inequality constraints for these branches are constructed based on a unified global error. Therefore, according to the global approximation theorem, each accepted node contributes to reducing the overall residual of the model. The pseudocode for TF-SCN is shown in Algorithm 1.
**Algorithm 1:** TF-SCN**Input:** Training dataset $D=\{(x_{i},y_{i})\}$; L_max_ is the maximum number of nodes in the hidden layer across both branches; maximum candidate trials T_max_; residual tolerance $E=[e_{1},e_{2},\cdots ,e_{C}]$; tolerable error *σ*; regularization coefficient λ; random parameter bound η.**Output:** Temporal parameters Θ_T_, frequency-sensitive parameters Θ_F_, output weight *β*, and prediction results.1. Initialize *H*_T_ = ∅, *H*_F_ = ∅, Θ_T_ = ∅, Θ_F_ = ∅, *L*_T_ = 1, *L*_F_ = 1.2. **while** (*L*_T_ < *L*_max_ or *L*_F_ < *L*_max_) and ||*E*|| > *σ* **do**:3.       Initialize candidate set *Φ* = ∅, Ω = ∅, t = 0;4.       **while** (*Θ*_T_ and *Θ*_F_ are empty) and t < T_max_ **do**:5.          Generate a random number *r* on the interval [0, 1];6.          Solve the sequence value: $\mu_{L}=(1-r)/(1+L_{T}+L_{F})$;7.          Generate $w_{t}^{T}$, $b_{t}^{T}$ randomly in [−*η*, *η*];8.          Generate $w_{t}^{F}$, $b_{t}^{F}$ randomly in [−*η*, *η*];9.          Construct temporal feature representation for the candidate node according to Equation (2);10.           Construct frequency feature representation for the candidate node according to Equation (3);11.           Calculate $\varepsilon_{q}^{T}$ and $\varepsilon_{q}^{F}$ according to Equation (8);12.           **if** $\min\left(\varepsilon_{1}^{T},\varepsilon_{2}^{T},\cdots ,\varepsilon_{C}^{T}\right)>0$:13.             Append $\left(w_{t}^{T},b_{t}^{T}\right)$ to the set *Φ*;14.             *L*_T_ = *L*_T_ + 1;15.           **end if**16.           **if** $\min\left(\varepsilon_{1}^{F},\varepsilon_{2}^{F},\cdots ,\varepsilon_{C}^{F}\right)>0$:17.             Append $\left(w_{t}^{F},b_{t}^{F}\right)$ to the set Ω;18.             *L*_F_ = *L*_F_ + 1;19.           **end if**20.           t = t + 121.      **end while**22.   Add $\left(w_{t}^{T},b_{t}^{T}\right)$ and $\left(w_{t}^{F},b_{t}^{F}\right)$ into Θ_T_, Θ_F_;23.   Solve *β* according to Equation (7);24.   Update residual matrix based on Equation (6);25.**end while**

## 3. Experimental Design

### 3.1. Data Preparation

**Figure 2:**
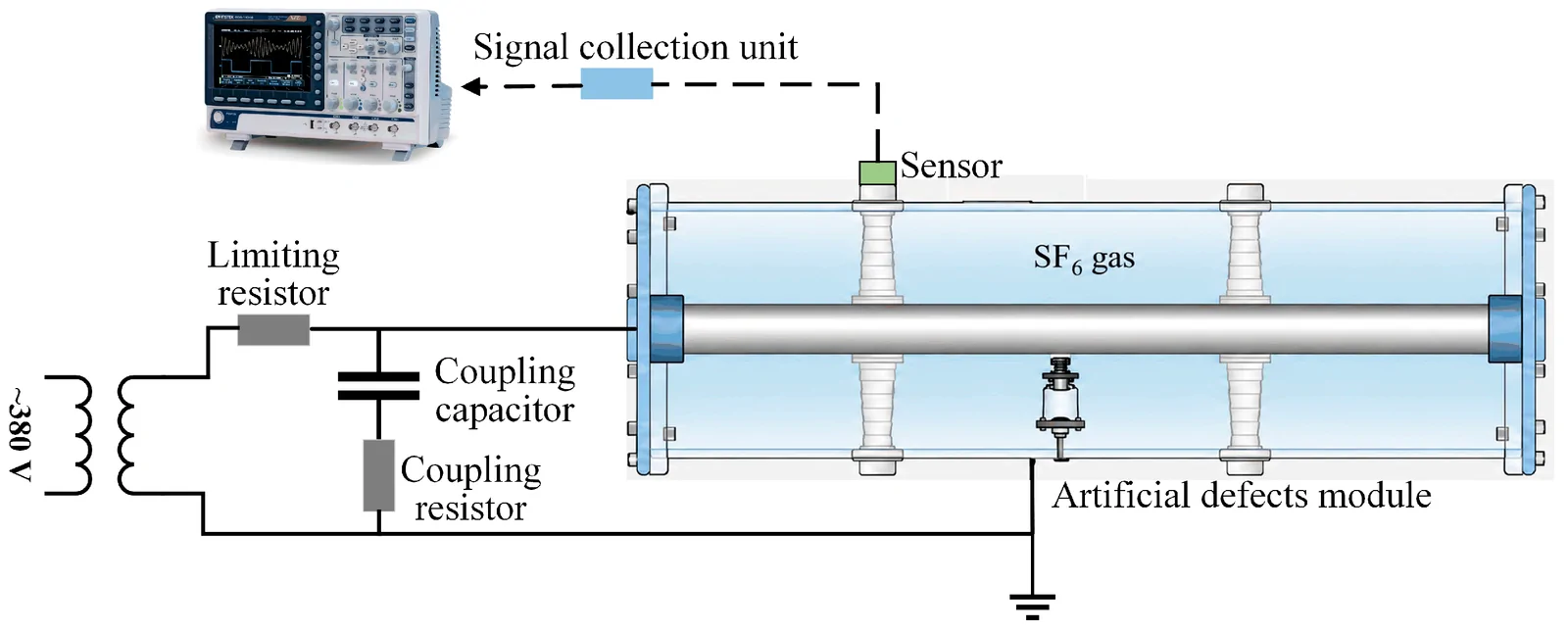
Schematic diagram of the GIS PD simulation platform.

The PD data presented in this study were collected from a laboratory GIS PD platform. The principle of the platform is shown in Figure 2. The setup mainly consists of a power-frequency step-up power source, a coupling capacitor, a GIS simulation chamber, artificial defect models, UHF sensors, and a signal acquisition unit. During the experiment, a 380 V power-frequency supply was applied to the central conductor of the GIS chamber through the step-up device. The chamber was filled with SF6 gas at 0.4 MPa. Artificial insulation defects were placed inside the chamber to generate PD activity. UHF sensors installed outside the GIS chamber served as the front-end data acquisition nodes of the proposed intelligent sensing system, receiving the electromagnetic pulses caused by PD. The signals are amplified and then sent to the data acquisition system, where oscilloscope (DPO4104B, Tektronix, Beaverton, OR, USA) is used to record the transient signals, yielding PD UHF pulse-event data for different defect types. Combined with the subsequent TF-SCN algorithm, this hardware–software integration achieves automated and intelligent fault diagnosis for the GIS.

In this paper, we consider four typical types of insulation defects, including metal tips, voids in a basin-type insulator, floating potentials, and free metal particles. Table 1 shows the sample sizes of pulse events for various PD patterns, where the pulse data include pulse duration and amplitude. To facilitate subsequent model recognition of different PD modes, we encode the four types of PD events induced by insulation defects, as depicted in Table 1.

To construct a standard time-pulse sequence, a fixed window containing *d* pulse events is employed. This window is slid with a step size of s to generate the next sequence sample. In this paper, *d* and *s* are set to 128 and 32, respectively, to balance temporal information preservation and sample generation under limited-data conditions. The resulting number of temporal pulse samples is listed in Table 1. The dataset is split into training and testing sets at a ratio of 8:2.

**Figure 3:**
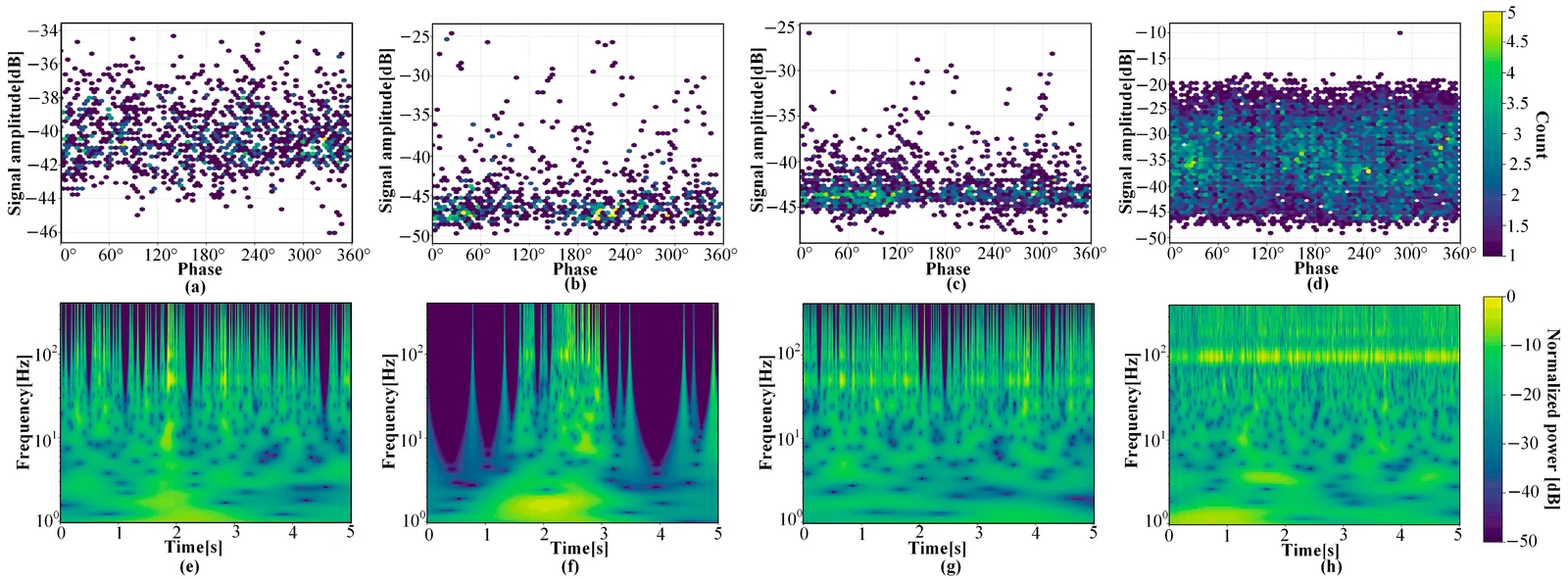
PRPD maps and time-frequency analysis of UHF pulse sequences. (**a**–**d**) show the PRPD maps generated by metal tip, void in basin-type insulator, floating potential, and metallic particle defects, respectively. (**e**–**h**) show the corresponding time-frequency maps of the four PD event sequences.

To investigate the separability of different PD patterns at temporal and frequency scales, PRPD maps and time-frequency maps based on continuous wavelet transform (CWT) are depicted for these data, as shown in Figure 3. The PRPD maps show that pulse sequences generated by different insulation defects exhibit significant differences in terms of phase clustering regions, discharge amplitude distributions, and pulse density. These differences indicate that pulse sequences contain recognizable temporal-scale features for distinguishing PD patterns. Furthermore, since the sequences consist of non-continuous UHF pulse data, the pulse events, which are unevenly spaced within each time window, are mapped onto a regular time grid to form a uniformly sampled sequence of PD events, facilitating CWT analysis. Specifically, for each pulse-event window ${\left\{(t_{i},s_{i})\right\}}_{i=1}^{N}$, where $t_{i}$ and $s_{i}$ are the occurrence time and signal amplitude of the PD event, respectively, the pulse occurrence times are first normalized to [0,1]. The interval is then divided into M uniform bins, and the uniformly sampled sequence is constructed by amplitude-weighted event accumulation:

(9) $$v_{m}=\sum_{i=1}^{N}s_{i}1\left(t_{i}\in B_{m}\right),\;\;\;\;m=1,2,\cdots ,M$$

where $B_{m}$ denotes the *m*-th time bin, and 1(·) is the indicator function. If no pulse event occurred in a bin, the corresponding value is set to zero. Subsequently, the resulting uniformly sampled amplitude sequence is employed for CWT-based time-frequency feature analysis.

Continuous wavelet transform (CWT) is a time-frequency analysis method that represents a signal by projecting it onto a family of scaled and translated mother wavelets. The scale factor determines the frequency resolution, while the translation factor preserves temporal localization information. Therefore, CWT is capable of characterizing transient and non-stationary features in PD UHF pulse sequences. In this study, the complex Morlet wavelet is selected as the mother wavelet due to its appropriate time-frequency localization capability for analyzing oscillatory transient signals.

Figure 3e–h shows the time-frequency maps of the UHF pulse sequence. The results reveal significant differences among the various PD patterns in terms of localized energy concentration regions, frequency-scale responses, and multiscale variation patterns. Notably, the results indicate that individual PD signals cannot be associated with a fixed frequency band, as shown in Figure 3e–h. Instead, they reflect macroscopic differences in pulse occurrence, energy concentration, and multi-scale response. For the metal tip defect, the sharp electrode produces a relatively stable and highly concentrated distorted electric field. The discharge process is therefore strongly modulated by the power-frequency voltage, leading to the continuous multi-scale energy distribution and voltage-related energy variation observed in Figure 3e. For the internal void defect, residual charge accumulation and release on the cavity wall introduce a memory effect, which increases the correlation between adjacent PD events and results in the pronounced local energy concentration shown in Figure 3f. For the floating potential defect, discharge is mainly governed by charge accumulation and sudden release on an isolated conductor, giving rise to intermittent impulsive responses and relatively concentrated high-frequency-scale energy in Figure 3g. For the free metallic particle defect, particle charging and motion dynamically alter the local electric field and discharge gap, making the pulse sequence more stochastic and producing a more dispersed time-frequency response in Figure 3h.

Overall, the time-frequency differences among the four PD patterns originate from variations in local electric-field structure, discharge path, charge accumulation–release behavior, and pulse randomness. These observations suggest that frequency-related information provides complementary discriminative cues beyond temporal features, thereby supporting the introduction of the PD pattern recognition model.

### 3.2. Evaluation Metrics

To evaluate the classification performance of different models for GIS PD pattern recognition, this paper uses accuracy (Acc), precision (P), recall (R), and F1-score (F1) as the main metrics. Confusion matrices are also utilized to show misclassification patterns between different defect classes. The metrics are defined as:

(10) $$\{\begin{array}{c} \begin{array}{c} \begin{array}{c} Acc=\frac{TP}{N}\\ P=\frac{1}{C}\sum_{c=1}^{C}\frac{{TP}_{c}}{{TP}_{c}+{FP}_{c}} \end{array}\\ R=\frac{1}{C}\sum_{c=1}^{C}\frac{{TP}_{c}}{{TP}_{c}+{FN}_{c}} \end{array}\\ F1=\frac{1}{C}\sum_{c=1}^{C}\frac{{TP}_{c}}{{TP}_{c}+{FP}_{c}}\cdot \frac{{TP}_{c}}{{TP}_{c}+{FN}_{c}} \end{array}$$

where ${TP}_{c}$, ${FP}_{c}$, ${TN}_{c}$, and $FN_{c}$ denote the true positives, false positives, true negatives, and false negatives of the c-th PD pattern, respectively. For the multi-class task in this paper, the reported precision, recall, and F1-score are calculated using the macro-averaging strategy. Each model was evaluated over 10 independent runs, and the mean and standard deviation of each metric are reported to reduce the influence of random initialization and data splitting.

### 3.3. Comparative Experiment Design

To verify the effectiveness of TF-SCN, 3 groups of experiments are designed: model comparison, ablation study, and noise robustness testing. Firstly, the proposed model is compared with several typical machine learning and neural-network models, including 1DCNN, BPNN, SVM, RVFL, KELM, and SCN. The 1DCNN model extracts one-dimensional convolutional features from the pulse sequence and completes classification through fully connected layers. BPNN uses a multilayer feedforward structure and updates its parameters by back-propagation. SVM serves as a classical kernel-based shallow classifier. RVFL and KELM are used as representative random learning models, while conventional SCN uses a single nonlinear activation function to build stochastic configuration nodes. To keep the comparison consistent, the hidden-layer size of the neural-network-related models is set to 128. The hyperparameter settings for the models are shown in Table A1 in Appendix A. An ablation study is then conducted to examine the contribution of the frequency-sensitive branch in TF-SCN. This paper constructs FFT-SCN based on the proposed model architecture. In this model, the input frequency features are preprocessed by FFT in the frequency-sensitive branch, and the activation function of this branch is replaced with tanh.

Since UHF PD signals in real-world operating conditions might be affected by sensor noise and on-site electromagnetic interference, Gaussian white noise is added to the raw sequences to construct datasets with different signal-to-noise ratios (SNR). To ensure consistency in sample size across different noise levels, noise is added to each segmented sequence rather than to the complete raw sequence.

## 4. Results and Discussion

### 4.1. Comparison with Conventional Models

**Figure 4:**
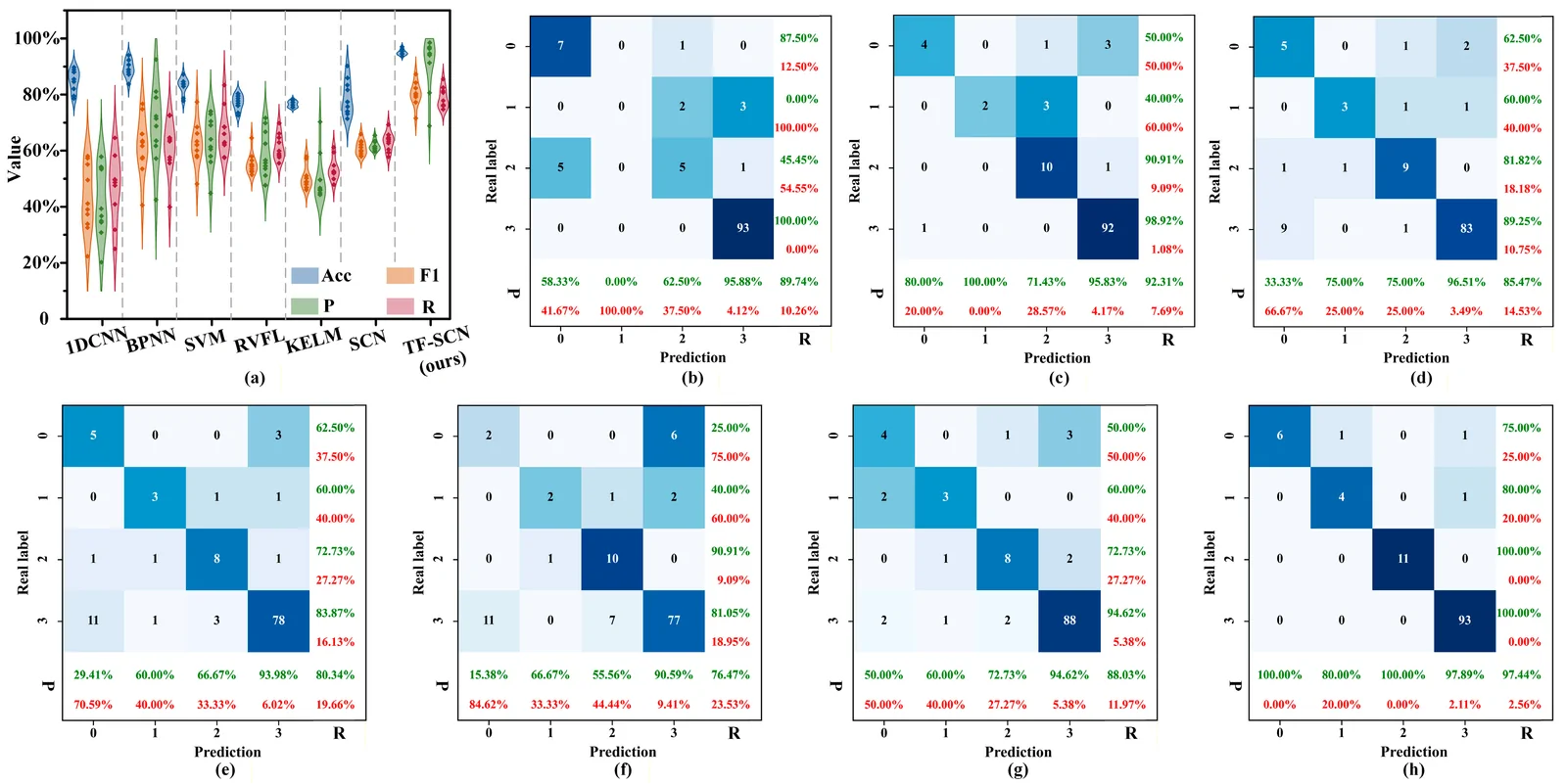
Performance comparison and confusion matrices of different models. (**a**) shows the box plots of performance metrics. (**b**–**h**) show the confusion matrices of 1DCNN, BPNN, SVM, RVFL, KELM, SCN, and the proposed model. Green values indicate the class-wise recall, precision, and overall accuracy, while red values represent the corresponding error rates.

To evaluate the advantage of the proposed model, TF-SCN is compared with typical neural-network and machine-learning methods. The results are shown in Table 2 and Figure 4a. The results show that TF-SCN achieves the best performance on all metrics. Its Acc, F1, P, and R reached 93.68%, 80.15%, 91.22%, and 78.70%, respectively. Compared with SCN, the proposed model improved Acc, F1, P, and R by 12.70%, 19.10%, 29.43%, and 15.54%. This indicates that the dual-branch heterogeneous feature representation substantially enhances the ability of SCN to classify PD patterns. According to Table 2, the baseline models show different limitations. Although 1DCNN achieves an Acc of 85.04%, its F1-score is only 42.63%, suggesting imbalanced recognition among classes and weak performance on some patterns. BPNN and SVM performed reasonably well on some metrics, but their F1 and R values are still clearly lower than those of TF-SCN. This suggests that conventional shallow models and standard neural networks have difficulty mining the complex nonlinear features in PD event sequences. RVFL and KELM show weaker overall performance, reflecting the limited representation ability of fully random mapping or kernel-based mapping in this task. SCN outperforms RVFL and KELM, but it is still limited by its single nonlinear activation function, which prevents it from simultaneously representing temporal and frequency-sensitive information. Moreover, TF-SCN achieves statistically significant improvements over all baseline models across different evaluation metrics (all *p*-values < 0.01), according to Table A2 in Appendix A.

TF-SCN retains the supervisory random configuration and output weight solution of SCN, while incorporating a conventional nonlinear temporal branch and a sine–cosine harmonic frequency-sensitive branch. Through hidden feature concatenation, this model more comprehensively captures the differences in partial discharge defect patterns in terms of temporal distribution, amplitude variation, and frequency-sensitive response. This explains its superior accuracy and overall recognition performance.

Figure 4b–h present the confusion matrices of the different models on the PD recognition task. The baseline models show varying degrees of class confusion, especially in the minority classes, namely class 0 and class 1. Under this condition, 1DCNN exhibits poor recognition performance for Class 1 samples. BPNN improves the recognition of class 2 and class 3, but its R values for class 0 and class 1 remained low. SVM, RVFL, and KELM show obvious confusion between class 0 and class 3, indicating that shallow classifiers and stochastic learning models struggle to construct reliable decision boundaries for complex PD event sequences. Overall, the proposed model achieves superior classification performance across different PD patterns compared with the baseline methods. Nevertheless, its recognition performance is still affected by the imbalance in the sample sizes across classes. Since the inequality-based supervision mechanism in SCN relies on overall residual information to select hidden nodes, the majority classes exert greater influence on the residuals, leading to comprehensive feature learning for the majority classes, while the representational capacity for the minority classes is limited.

SCN represents an improvement over RVFL and KELM, but its single nonlinear random basis mapping still limits its ability to recognize certain classes. In contrast, TF-SCN yields the fewest misclassified samples, achieving recall values of 100% for both Classes 2 and 3. These results confirm that the heterogeneous fusion of time-domain and frequency-sensitive features can enhance the separability of different PD patterns and achieve more balanced classification.

### 4.2. Ablation Study

To verify the effectiveness of the branching structure in TF-SCN, we conduct an ablation study. As shown in Table 3, when the model contains only the temporal SCN branch, its Acc and F1 are 80.98% and 61.05%. This indicates that conventional SCN captures some temporal features in the sequences, but its discriminative ability remains limited by a single nonlinear random basis function. Similarly, the model adopting only the frequency-sensitive SCN branch exhibits an Acc of 70.26%. This demonstrates that, although sine-cosine harmonic nodes are capable of detecting frequency characteristics, they alone cannot fully describe the temporal structure and amplitude distribution of PD event sequences.

FFT-SCN utilizes the fast Fourier transform (FFT) to preprocess the pulse sequence and inputs the spectral data into the frequency-sensitive branch. Its Acc is 72.05%, slightly higher than that of the frequency-sensitive branch alone, but much lower than that of TF-SCN. Different PD pulse sequences exhibit varying distributions of frequency components; FFT-based preprocessing may struggle to provide a unified and universally effective set of frequency features for all PD modes. In other words, the fixed spectral components obtained via FFT consistently fail to correspond to the most discriminative frequency-related information for different PD modes. By contrast, the frequency-sensitive branch in TF-SCN directly selects effective harmonic features through residual-guided random configuration. Specifically, it introduces sine–cosine harmonic nodes in the hidden layer and adaptively explores effective frequency-related representations through random configuration and node selection based on residual constraints. This enables the model to capture useful implicit frequency-sensitive information from the original PD sequences, thereby achieving better recognition performance.

**Figure 5:**
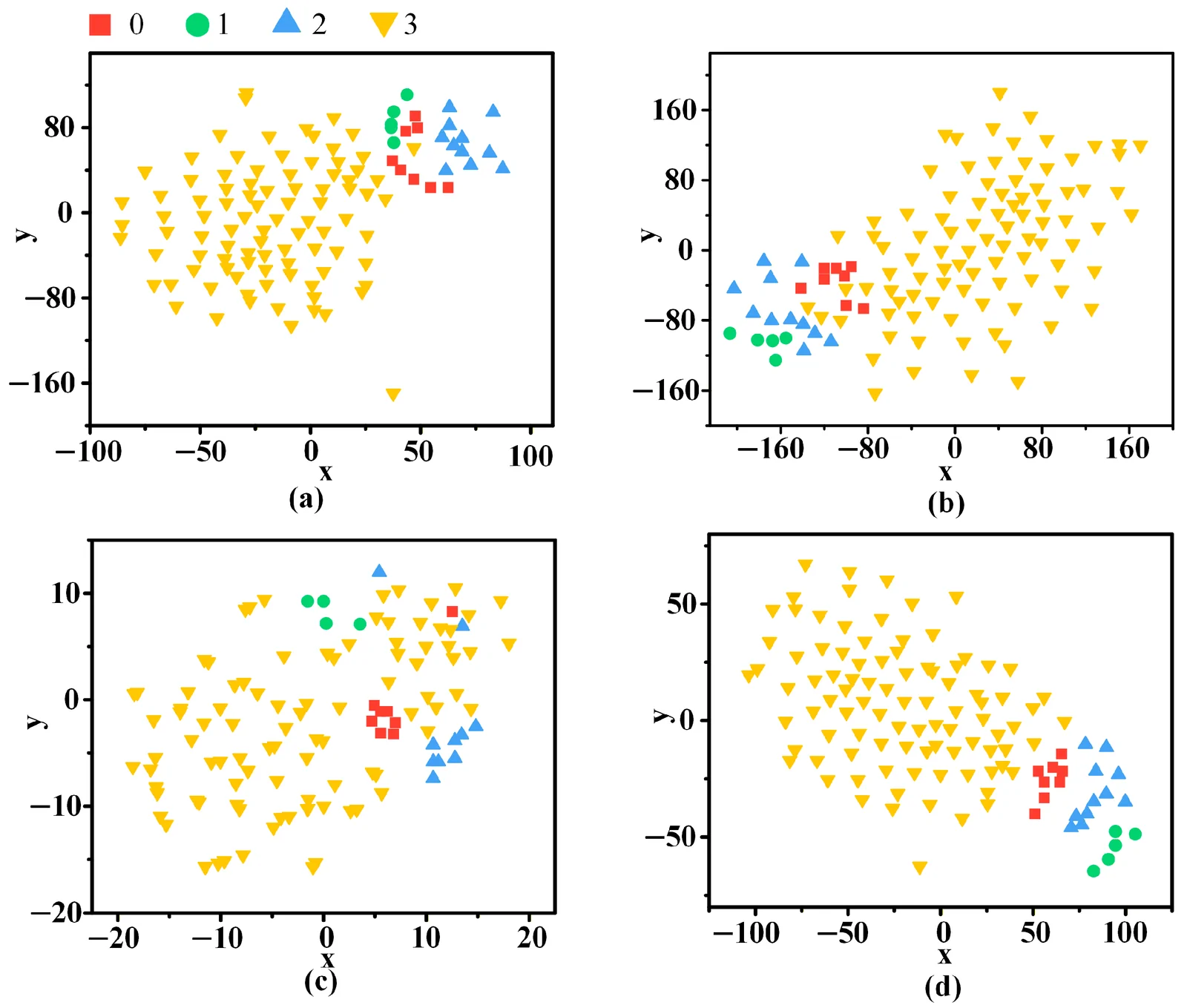
Feature Distribution Maps Based on t-SNE. (**a**,**b**) represent the feature maps of the model containing temporal and frequency-sensitive branches, respectively; (**c**,**d**) show the feature maps of FFT-SCN and TF-SCN, respectively.

To further evaluate the feature representation capability of different models, t-SNE visualization is employed for the hidden-layer features, as shown in Figure 5. The temporal branch in Figure 5a exhibits noticeable inter-class overlap, particularly for the low-abundance Classes 0 and 1, indicating that temporal information alone is insufficient to distinguish PD patterns. In comparison, the frequency-sensitive branch in Figure 5b improves the separation of these classes to some extent, demonstrating the complementary discriminative information provided by harmonic mapping. In Figure 5c, FFT-SCN still shows relatively dispersed distributions for Classes 0 and 1, suggesting that explicit FFT-based spectral preprocessing has limited capability in representing these low-abundance classes. By contrast, TF-SCN in Figure 5d produces more compact intra-class distributions and clearer inter-class boundaries, especially for Classes 0 and 1. These observations provide qualitative evidence that the proposed harmonic mapping captures complementary frequency-sensitive information more effectively than FFT preprocessing and that temporal–frequency heterogeneous fusion improves the representation of PD patterns.

### 4.3. Noise Robustness Comparison

**Figure 6:**
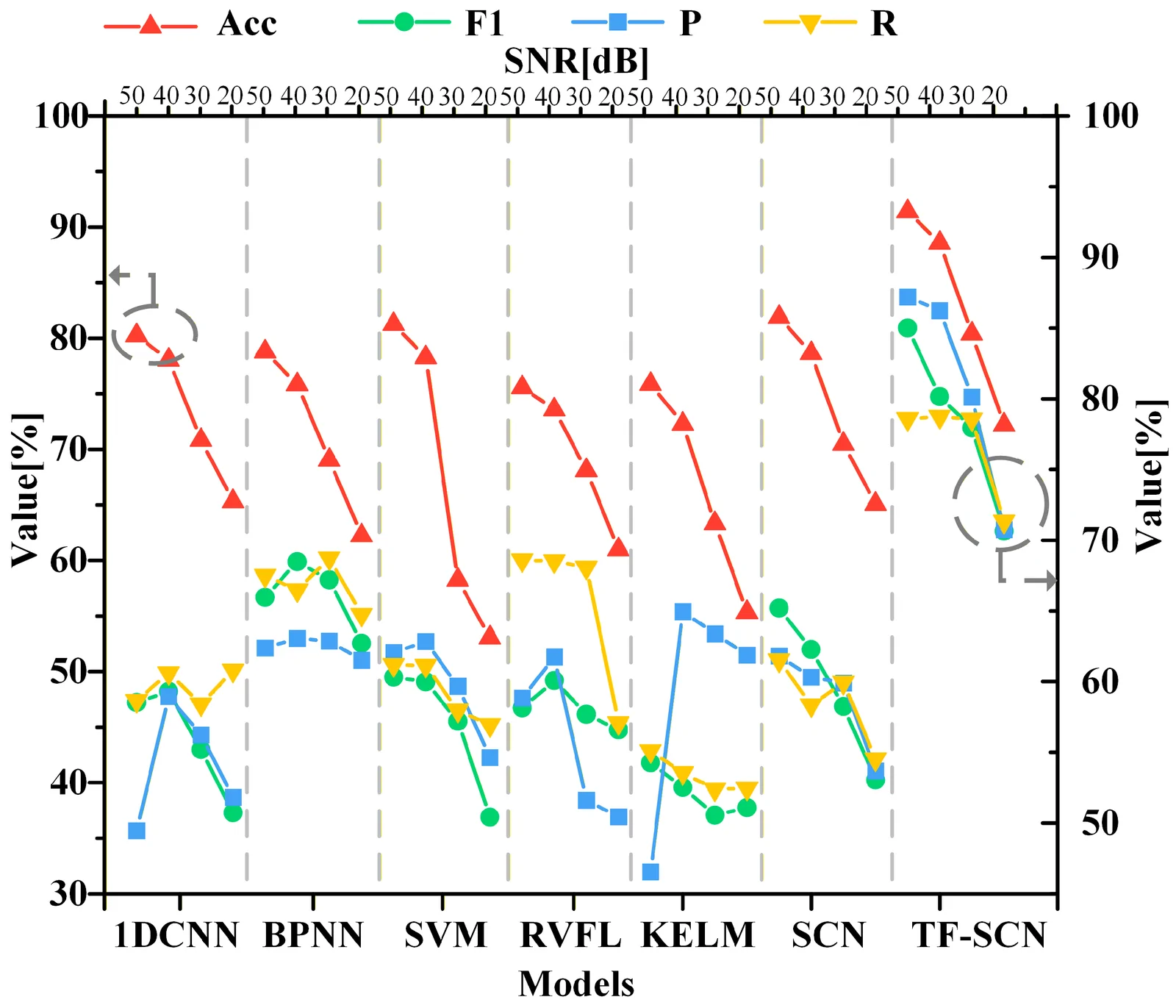
Performance curves of different models under different noise levels.

To further test the robustness of the models, Gaussian white noise of varying intensities was introduced into the dataset. Figure 6 shows the performance trends of all models across an SNR range from 50 to 20 dB. As the SNR decreases, the performance of all models generally declines. This is understandable, as noise weakens the amplitude distribution, local pulse structure, and frequency characteristics of the sequences, leading to reduced discrimination among different modes. Even under these degraded conditions, TF-SCN consistently achieves optimal performance. Across all SNR levels, its Acc, F1, P, and R curves remain superior to those of other models.

Table 4 presents the detailed results. At an SNR of 50 dB, TF-SCN achieves Acc, F1, P, and R values of 91.41%, 85.01%, 87.20%, and 78.58%, respectively. Compared with the values of the best baseline model at the same noise level, these metrics are improved by 9.51%, 19.05%, 24.82%, and 9.99%, respectively. Even at the lower SNR of 20 dB, TF-SCN still achieves the best performance across all 4 metrics.

Compared with conventional SCN, TF-SCN improved Acc by 9.51%, 9.92%, 9.93%, and 7.13% at 50, 40, 30, and 20 dB, respectively. Its F1-score improved by 19.81%, 17.88%, 19.69%, and 17.61% over the same SNR range. These results show that a single nonlinear stochastic configuration node has limited representation ability under noise disturbance. By fusing the temporal branch with the harmonic frequency-sensitive branch, TF-SCN preserves more complementary discriminative information in PD event sequences and achieves more stable recognition as the noise level increases.

## 5. Conclusions

To tackle the challenge of adequately representing temporal and frequency features in PD UHF pulse sequences, this paper proposes TF-SCN for GIS PD pattern recognition. Based on the supervised stochastic configuration framework from SCN, the model constructs a conventional nonlinear temporal branch and a paired sine–cosine harmonic frequency-sensitive branch. The hidden-layer features of the two branches are then concatenated to form a heterogeneous temporal-harmonic joint feature space, thereby enhancing the extraction of discriminative time-frequency information from PD UHF pulse sequences. To verify the superiority of TF-SCN, we compare it with six conventional models on the PD UHF dataset collected from the GIS PD simulation platform. The results show that TF-SCN achieves Acc, F1, P, and R values of 93.68%, 80.15%, 91.22%, and 78.70%, respectively, outperforming 1DCNN, BPNN, SVM, RVFL, KELM, and conventional SCN. The ablation study further confirmed the complementarity between the temporal branch and the frequency-sensitive branch, indicating that simple FFT preprocessing fails to replace hidden-layer harmonic feature mapping. In noise-robustness testing, the performance of these models declined as SNR decreased, but TF-SCN consistently maintained the best performance at SNRs ranging from 50 to 20 dB. Notably, for sequences with an SNR of 20 dB, TF-SCN achieved Acc and F1 of 72.21% and 70.65%, respectively. These results show that TF-SCN improves both recognition accuracy and robustness, offering an effective lightweight method for intelligent GIS PD diagnosis.

Although the proposed TF-SCN provides an effective solution for PD pattern recognition with a limited number of training samples, some challenges remain for practical deployment. The imbalance among different PD categories and the discrepancy in feature distributions between laboratory and field conditions may affect model generalization. Future studies will investigate more robust learning strategies, such as imbalance-aware and cross-domain adaptation methods, to further improve the reliability of intelligent GIS PD diagnosis under complex operating environments.

## Figures and Tables

**Table 1:** Data scale and coding of different PD patterns.

Insulation Defects	Code	Total Pulse Events	Sequential Pulse Samples	Training	Testing
Metal tips	0	1365	39	31	8
voids in basin-type insulator	1	950	26	21	5
floating potentials	2	1810	53	42	11
free metal particles	3	14,985	465	372	93

**Table 2:** Performance comparison of different models.

Model	Acc [%]	F1 [%]	P [%]	R [%]
1DCNN	85.04 ± 3.9%	42.63 ± 12.0%	41.57 ± 12.5%	47.42 ± 12.1%
BPNN	89.32 ± 3.0%	61.46 ± 10.4%	69.0 ± 13.9%	61.14 ± 9.5%
SVM	83.16 ± 2.8%	62.36 ± 7.6%	63.15 ± 9.0%	66.59 ± 8.2%
RVFL	77.18 ± 2.7%	55.35 ± 3.8%	58.99 ± 8.2%	61.21 ± 4.5%
KELM	76.58 ± 1.1%	50.39 ± 4.0%	49.73 ± 8.4%	53.49 ± 4.0%
SCN	80.98 ± 6.3%	61.05 ± 2.6%	61.79 ± 1.7%	63.16 ± 3.3%
TF-SCN	93.68 ± 1.0%	80.15 ± 4.2%	91.22 ± 9.3%	78.70 ± 3.6%

**Table 3:** Ablation study results.

Model		Acc	F1	P	R
T	F				
√	×	80.98 ± 6.3%	61.05 ± 2.6%	61.79 ± 1.7%	63.16 ± 3.3%
×	√	70.26 ± 4.2%	59.28 ± 5.4%	59.22 ± 4.6%	58.18 ± 7.2%
FFT-SCN		72.05 ± 4.4%	60.58 ± 5.1%	58.35 ± 4.1%	60.72 ± 7.1%
√	√	93.68 ± 1.0%	80.15 ± 4.2%	91.22 ± 9.3%	78.70 ± 3.6%

**Table 4:** Performance of different models under different SNR levels.

SNR [dB]	Model	Acc [%]	F1 [%]	P [%]	R [%]
50	1DCNN	80.25 ± 2.5	58.53 ± 2.7	49.45 ± 2.4	58.65 ± 2.6
	BPNN	78.78 ± 5.1	65.96 ± 6.2	62.38 ± 4.6	67.50 ± 5.4
	SVM	81.27 ± 4.6	60.31 ± 4.9	62.07 ± 4.6	61.19 ± 4.6
	RVFL	75.57 ± 3.4	58.14 ± 3.9	58.83 ± 3.2	68.59 ± 3.7
	KELM	75.87 ± 6.3	54.26 ± 6.6	46.55 ± 4.9	55.10 ± 6.5
	SCN	81.90 ± 7.1	65.20 ± 7.9	61.82 ± 8.7	61.52 ± 7.4
	TF-SCN	91.41 ± 4.0	85.01 ± 4.5	87.20 ± 4.1	78.58 ± 4.3
40	1DCNN	78.05 ± 3.4	59.30 ± 3.4	58.95 ± 2.6	60.61 ± 3.2
	BPNN	75.84 ± 4.2	68.48 ± 4.3	63.07 ± 3.5	66.47 ± 4.1
	SVM	78.27 ± 4.7	59.98 ± 5.3	62.84 ± 5.0	61.14 ± 5.0
	RVFL	73.57 ± 4.0	60.08 ± 4.4	61.75 ± 3.5	68.53 ± 4.2
	KELM	72.29 ± 3.5	52.52 ± 4.5	64.95 ± 3.1	53.54 ± 3.7
	SCN	78.65 ± 3.4	62.27 ± 4.7	60.31 ± 3.3	58.34 ± 3.8
	TF-SCN	88.57 ± 4.3	80.15 ± 4.8	86.24 ± 4.4	78.74 ± 4.7
30	1DCNN	70.84 ± 3.4	55.19 ± 3.3	56.24 ± 2.8	58.40 ± 2.9
	BPNN	69.05 ± 6.3	67.20 ± 8.2	62.86 ± 5.8	68.74 ± 6.8
	SVM	58.25 ± 7.1	57.20 ± 7.8	59.67 ± 7.8	57.97 ± 7.3
	RVFL	68.10 ± 4.8	57.69 ± 5.4	51.61 ± 4.1	68.08 ± 5.1
	KELM	63.33 ± 6.6	50.55 ± 6.2	63.38 ± 5.4	52.39 ± 6.7
	SCN	70.48 ± 4.9	58.25 ± 5.2	59.91 ± 6.2	59.94 ± 4.8
	TF-SCN	80.41 ± 4.4	77.94 ± 5.1	80.12 ± 4.5	78.56 ± 4.7
20	1DCNN	65.32 ± 3.6	50.72 ± 3.6	51.81 ± 2.9	60.79 ± 3.5
	BPNN	62.24 ± 4.3	62.71 ± 5.9	61.52 ± 4.1	64.74 ± 4.7
	SVM	53.05 ± 6.9	50.41 ± 7.4	54.63 ± 7.8	56.94 ± 7.0
	RVFL	60.98 ± 4.7	56.59 ± 5.0	50.45 ± 3.9	57.07 ± 4.8
	KELM	55.33 ± 5.2	51.08 ± 6.3	61.88 ± 9.2	52.46 ± 5.2
	SCN	65.08 ± 6.0	53.04 ± 6.2	53.68 ± 7.3	54.52 ± 6.1
	TF-SCN	72.21 ± 4.6	70.65 ± 5.2	70.75 ± 4.4	71.33 ± 4.9

## Data Availability

The original contributions presented in this study are included in the article. Further inquiries can be directed to the corresponding author.

## Appendix A

**Table A1:** Hyperparameter configuration table.

Parameter	Value	Parameter	Value
d	128	T_max_	50
s	32	λ	1
L_max_	128	*η*	1

**Table A2:** *p*-values for TF-SCN compared to other baseline models across various metrics.

Comparison	Acc	F1	P	R
TF-SCN vs. 1DCNN	1.31 × 10^−4^	5.70 × 10^−6^	4.77 × 10^−6^	2.27 × 10^−5^
TF-SCN vs. BPNN	1.23 × 10^−3^	2.86 × 10^−4^	4.00 × 10^−3^	4.30 × 10^−4^
TF-SCN vs. SVM	9.00 × 10^−7^	6.91 × 10^−5^	7.22 × 10^−5^	1.37 × 10^−3^
TF-SCN vs. RVFL	3.52 × 10^−8^	1.11 × 10^−9^	1.39 × 10^−5^	2.42 × 10^−7^
TF-SCN vs. KELM	7.16 × 10^−12^	4.65 × 10^−8^	7.42 × 10^−6^	2.13 × 10^−8^
TF-SCN vs. SCN	1.81 × 10^−4^	5.55 × 10^−7^	4.81 × 10^−6^	4.93 × 10^−6^
